# Nanostructured Transition Metal Oxides on Carbon Fibers for Supercapacitor and Li-Ion Battery Electrodes: An Overview

**DOI:** 10.3390/ijms25158514

**Published:** 2024-08-04

**Authors:** Andrés González-Banciella, David Martinez-Diaz, María Sánchez, Alejandro Ureña

**Affiliations:** 1 Materials Science and Engineering Area, Escuela Superior de Ciencias Experimentales y Tecnología, Universidad Rey Juan Carlos, C/Tulipán s/n, 28933 Mostoles, Spain; andres.banciella@urjc.es (A.G.-B.); david.martinez.diaz@urjc.es (D.M.-D.); alejandro.urena@urjc.es (A.U.); 2 Instituto de Investigación de Tecnologías para la Sostenibilidad, Universidad Rey Juan Carlos, C/Tulipán s/n, 28933 Mostoles, Spain

**Keywords:** nanomaterials, carbon fiber, transition metal oxides, Li-ion battery, supercapacitors

## Abstract

Nowadays, owing to the new technological and industrial requirements for equipment, such as flexibility or multifunctionally, the development of all-solid-state supercapacitors and Li-ion batteries has become a goal for researchers. For these purposes, the composite material approach has been widely proposed due to the promising features of woven carbon fiber as a substrate material for this type of material. Carbon fiber displays excellent mechanical properties, flexibility, and high electrical conductivity, allowing it to act as a substrate and a collector at the same time. However, carbon fiber’s energy-storage capability is limited. Several coatings have been proposed for this, with nanostructured transition metal oxides being one of the most popular due to their high theoretical capacity and surface area. In this overview, the main techniques used to achieve these coatings—such as solvothermal synthesis, MOF-derived obtention, and electrochemical deposition—are summarized, as well as the main strategies for alleviating the low electrical conductivity of transition metal oxides, which is the main drawback of these materials.

## 1. Introduction

In recent years, energy-storage devices have become an essential part of our technology. In this way, the markets for both batteries and supercapacitors have continued to grow [1,2]. In the case of the battery market, valued in 2020 at USD 12.9 billion, it is expected to grow by 15.8% in terms of its compound annual growth rate (CAGR) until at least 2030 [3]. Despite the fact that there are several kinds of batteries within this market, not only are Li-ion batteries the most popular ones, but they are also expected to increase the most in market volume in absolute terms [3]. On the other hand, supercapacitors are said to be the great revolution in the energy-storage field. This market, valued at USD 4.46 billion in 2023, is expected to continue expanding by a 14.1% CAGR until 2030 [4]. However, the new central role of both markets has been to meet not only the increased demand but also the technical requirements of these devices. New supercapacitors and batteries, with increasingly higher power and energy densities, are needed to meet the requirements of technological advancements [5]. In fact, not only are electrochemical performance requirements on the rise, but so is the development of energy-storage devices with new functionalities, such as flexibility or the ability to act as a structural component. Regarding flexible energy-storage devices, the growing demand is driven by the significant expansion of the flexible electronic device market in recent years via smartphones and tablets [6]. Flexibility has been extended to new devices, especially in the field of monitoring biomedical signals, thanks to their easy wearability [7,8]. On the other hand, structural energy-storage devices are particularly interesting in terms of improving the performance of new electric vehicles through the combination of lightness and good mechanical behavior [9].

In this way, the electric vehicle market is presented as one of the largest consumers of these types of energy-storage devices, being estimated at EUR 518.000 million in 2023, and is expected to witness an annual growth rate of 14.5% from 2024 to 2030 [10]. Currently, electric vehicles have emerged as a major alternative to traditional gasoline or diesel combustion vehicles, being widely promoted both politically and industrially to curb the high levels of pollution in big cities [11] and the depletion of oil reserves [12,13]. However, electric vehicles have not yet fully surpassed combustion vehicles due, among other things, to their lower performance, especially in terms of autonomy, and higher weight (around 33% higher) [14]. To mitigate this problem, one of the most efficient alternatives is a reduction in the total vehicle mass, allowing the same amount of energy to propel a vehicle for a larger number of kilometers. Here is where the concept of multifunctionality gains importance, developing structures that, in addition to having sufficient structural properties for the desired applications, can also store energy. In this way, the total or partial need for conventional batteries or supercapacitors could be reduced, decreasing the vehicle’s final weight and thus increasing its autonomy [15].

**Figure 1:**
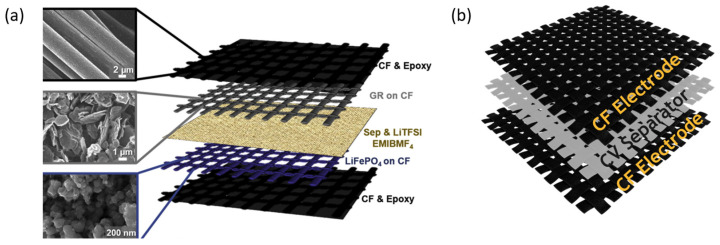
(**a**) A reported structural battery scheme [21]. Copyright Elsevier, 2020. (**b**) Multifunctional material for energy-storage-layers scheme.

The development of multifunctional materials that can also store energy while presenting good structural properties has been primarily focused on the development of composite materials [15]. The popularity of composite materials for such applications is mainly due to the suitability of carbon fiber as a mechanical reinforcement and as a substrate for electrodes. Its excellent structural properties, such as high elastic modulus and high tensile strength, make carbon fiber fabric an excellent candidate for the development of structural composite materials [16]. Additionally, its high electrical conductivity also allows people to use it as a current collector for application in supercapacitors and batteries [17]. On the other hand, the small diameter of the carbon fibers also enables the development of flexible composite materials [16,18]. In this way, multifunctional composite materials, developed for energy-storage purposes, are commonly based on carbon fiber electrodes; separators made of insulating fabric, usually fiberglass; and a polymeric matrix that acts as an electrolyte [19,20,21], as shown in the schematics in Figure 1a,b.

However, the development of this type of material faces two main challenges. The first one is the development of solid electrolytes, where high ionic mobility is a requirement for both supercapacitor and battery electrolyte development. However, as the stiffness increases, being necessary to obtain structural composites, the ionic mobility in the polymeric matrix decreases [15,22,23]. The second challenge is the functionalization of carbon fiber to act as a functional electrode [24]. Despite the fact that unmodified carbon fiber has been reported on several occasions to be a structural lithium-ion battery anode [22], its performance can be significantly increased through coating with other materials [24]. These coatings are necessary in the case of the development of cathodes [25,26,27,28] or supercapacitor electrodes [29,30,31].

The challenges in developing solid-state energy-storage systems, along with the trade-off between stiffness and ionic conductivity, necessitate the definition of new parameters to evaluate the multifunctionality of the materials being developed. In 2011, O’Brien et al. [32] defined multifunctional efficiency ($\eta_{mf})$ as the sum of the ratios between the specific energy (${\bar{w}}_{mf}$) and the elastic modulus (${\bar{E}}_{mf}$) of the multifunctional material with the specific energy ($\bar{w})$ and the elastic modulus ($\bar{E})$ of a conventional energy-storage system and a conventional structural material, respectively (Equation (1)). This definition was later expanded in 2015 by Snyder et al. [33], redefining these ratios to incorporate the shear modulus and specific power (Equations (2) and (3)).

(1) $$\eta_{mf}=\eta_{s}+\eta_{e}=\frac{{\bar{E}}_{mf}}{\bar{E}}+\frac{{\bar{w}}_{mf}}{\bar{w}}$$

(2) $$\eta_{s}=\min\left(\frac{{\bar{E}}_{mf}}{\bar{E}},\frac{{\bar{G}}_{mf}}{\bar{G}}\right)$$

(3) $$\eta_{e}=\min\left(\frac{{\bar{w}}_{mf}}{\bar{w}},\frac{{\bar{P}}_{mf}}{\bar{P}}\right)$$

Thus, a multifunctional composite material must satisfy $\eta_{mf}>1$ to be of real interest to the industry. However, actually, the most advanced multifunctional materials have barely achieved multifunctional efficiencies of 0.5 [22].

## 2. TMOs for Energy-Storage Applications

As will be more extensively discussed in the next sections, TMOs have been widely reported for Li-ion battery anode and supercapacitor electrode applications. Nevertheless, the requirements for these energy-storage components, the TMO advantages and disadvantages for these purposes, and the strategies to address the drawbacks should be clarified in order to develop a comprehensive state of the art.

### 2.1. TMOs as Supercapacitor Electrodes

**Figure 2:**
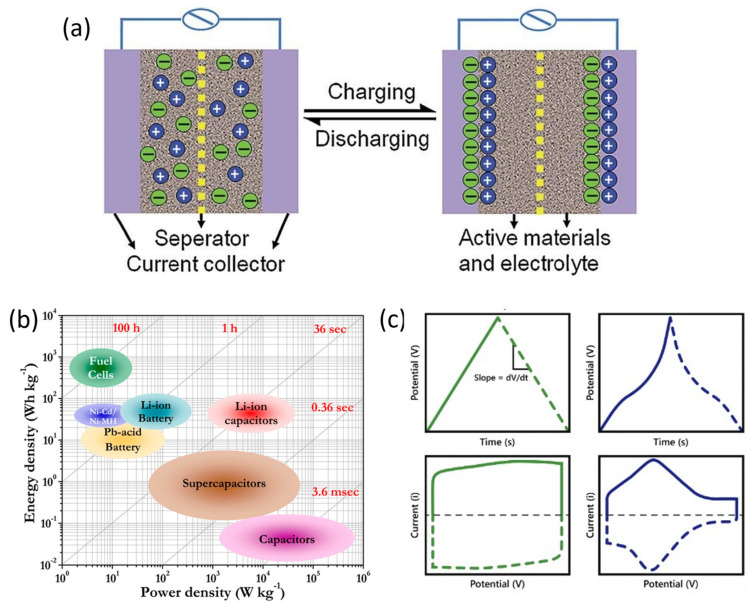
(**a**) Supercapacitor charge−discharge mechanism [43]. Copyright Elsevier, 2013. (**b**) Ragone plot. Reproduced from [44]. (**c**) Typical GCD (up) and CV (down) curves of EDLC supercapacitors (green) and pseudocapacitors (blue). Reproduced from [45].

Supercapacitors are energy-storage devices in which the primary mechanism of energy storage is electrostatic interactions. Supercapacitors consist of three main components: two electrodes connected by an electrical circuit, and a dielectric medium between them called electrolyte. During charging cycles, an applied potential polarizes the electrodes, causing charges within the electrolyte to align on the surface of the electrodes, forming an electric double-layer capacitor (EDLC) through electrostatic interactions [34]. This arrangement is schematically illustrated in Figure 2a. The charge stored in this EDLC increases linearly with the potential. Moreover, the addition of a new charge element to the EDLC involves additional electrical work due to previously accumulated elements having the same charge sign [35]. On the other hand, during discharge, the external potential ceases, and electric current flows from the negatively charged electrode to the positive one, while charges in the electrolyte homogenize through diffusion. As a result, the potential between electrodes decreases linearly as the charge storage decreases. Because of this linear dependence of the accumulated or released charge on potential, the concept of capacitance emerges. Capacitance (C) is defined as the change in charge (∆Q) with respect to the change in potential (∆V)—in other words, the slope of this linear relationship. Thus, considering the mass or volume of material (Π), the specific capacitance ($C_{s}$) is defined as Equation (4) [36]:

(4) $$C_{s}=\frac{∆Q}{∆V\Pi }$$

In an EDLC supercapacitor, the relationship between charge and potential mainly depends on the area of the electric double layer (A) and its thickness (d). Taking into account that the thickness may not be constant and the permittivity of the double layer ($\varepsilon_{dl}$) and vacuum ($\varepsilon_{0}$), the capacitance of the electrode (C) is defined as Equation (5) [37]:

(5) $$C=\int \frac{\varepsilon_{dl}\varepsilon_{0}}{d}A$$

Moreover, it is also necessary to quantify the energy that a device can accumulate or release, hence the concept of specific energy or energy density ($W_{s}$), which, considering the linear relationship between the charge and the potential, is given by [36] (Equation (6)):

(6) $$E_{s}=\frac{\int_{V_{0}}^{V}QdV}{\Pi }=\frac{1}{2}{C_{s}V}^{2}$$

On a technical level, it is not only important to consider the energy stored by the device, but it is also necessary to consider the power that can be supplied, that is, how quickly it can release that energy. Thus, the specific power or power density ($P_{s}$), considering the discharge time ($t_{dis}$), can be defined as Equation (7) [38]:

(7) $$P_{s}=\frac{E_{s}}{t_{dis}}$$

The specific power of supercapacitors is particularly high compared to other energy-storage systems such as batteries or fuel cells, as can be seen in the Ragone plot shown in Figure 2b. This is because the energy-storage mechanism is based on electrostatic interactions, weak interactions without charge transfer, that allow rapid desorption of ions from the electric double layer. However, this same weakness of the bonds results in these devices having lower stored energy compared to their aforementioned competitors [39,40].

Nevertheless, there is another mechanism by which a supercapacitor can store energy, known as pseudocapacitance, characteristic of pseudocapacitors. Pseudocapacitance involves faradaic reactions between the polarized electrode surface and ions in the electrolyte with partial charge transfer. Since these reactions take place at a specific potential, there is an increase in the accumulated charge at that potential. As a result, the relationship between accumulated charge and potential is not linear. Figure 2c shows the galvanostatic charge–discharge and cyclic voltammetry tests for comparison between EDLC supercapacitors and pseudocapacitors. In galvanostatic tests, the accumulation of charge at a specific potential due to pseudocapacitive reactions results in a plateau in the curve at that potential, while in cyclic voltammetry tests, these reactions appear as peaks [41]. This additional energy-storage mechanism provides additional capacitance that depends on the number of exchanged electrons (n) in the reaction and can be theoretically calculated as Equation (8) [42], where F is Faraday’s constant, M is the molar mass of the material, and $V_{0}$ is the potential window.

(8) $$C=\frac{nF}{MV_{0}}$$

During recent years, transition metal oxides (TMOs) have been extensively studied as materials for supercapacitor electrodes. The interest is driven by two fundamental reasons: the reactivity of these compounds to undergo pseudocapacitance reactions and their ease of synthesis in various nano-scale morphologies [42]. Both the presence of pseudocapacitance reactions and high values of surface area, resulting from the low dimensionality of these nanostructures, favor elevated specific capacitance values. However, the main drawback of TMOs for supercapacitor electrode applications is their low electrical conductivity [46,47,48]. This lack of electrical conductivity restricts the passage of electrons involved in chemical reactions, as well as the formation of the electric double layer. For this reason, several strategies to improve this aspect have been widely suggested [42,49]:

- Combination with high electrical conductivity materials. The combined use with other compounds that exhibit high electrical conductivity, such as carbon nanoplates, graphene oxide, reduced graphene oxide, carbon nanotubes [50,51], carbon black, MXenes, or conductive polymers [52,53].
- Sulfurization or selenization. These elements, which are softer Pearson bases than oxygen, polarize the metal bond less, reducing the band gap [54].
- Creation of oxygen vacancies. Oxygen vacancies lead to the presence of metal cations with a higher oxidation state within the crystalline structure, resulting in the appearance of discrete energy levels allowed in the band gap, facilitating the passage of electrons from the valence band to the conduction band [55,56].
- Doping with donor dopants. Doping with elements such as P, which promotes electrons to the conduction band [57].
- Preparation of multimetallic or multivalent TMOs. Similarly to the previous case, the presence of different cations leads to lower band gaps due to the greater richness of allowed energy levels [58,59].

### 2.2. TMOs as Li-Ion Batteries Anodes

Rechargeable batteries are energy-storage devices whose charge-storage mechanism is based on reversible faradaic reactions [35]. Li-ion batteries have been the most widely distributed and commercialized since they entered the market due to the small atomic radius of Li^+^ facilitating its diffusion. Moreover, the Li^+^/Li potential is quite low, −3.04 V versus a standard hydrogen electrode, resulting in a high potential that translates into high energy and power densities [60]. The energy density of batteries is higher than that achieved by supercapacitors [39], as can be observed in Figure 2b. This higher energy density is due to the storage through chemical reactions, which are more energetic than the electrostatic interactions characteristic of supercapacitors. However, this same difference also results in a lower power density due to the kinetic limitations of charge-transfer reactions [35].

**Figure 3:**
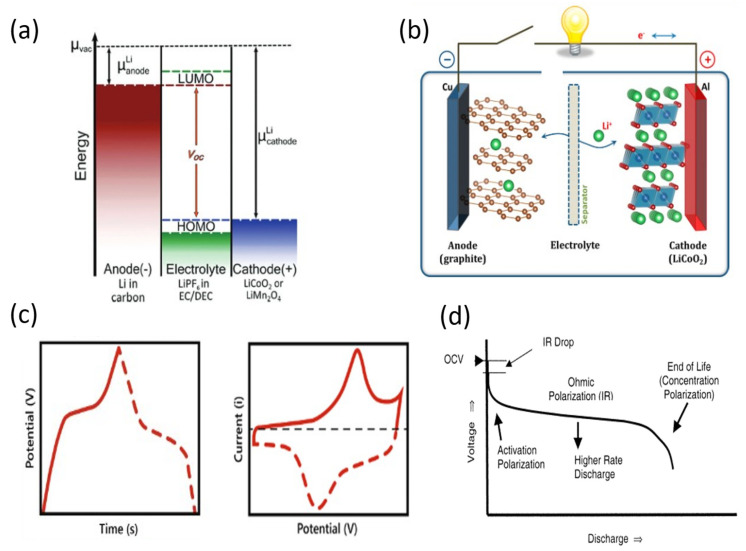
(**a**) Energy levels of Li−ion battery components. Reprinted with permission from [66]. Copyright 2013, American Chemical Society. (**b**) Li−ion battery scheme. Reprinted with permission from [62]. Copyright 2013, American Chemical Society. (**c**) Typical GCD (left) and CV (right) curves of Li−ion batteries [45]. (**d**) Voltage evolution during a discharge. Reprinted with permission from [64]. Copyright 2004, American Chemical Society.

Lithium-ion batteries consist of a negative electrode or anode, a positive electrode or cathode, and an electrolyte. As shown in Figure 3a, the Fermi level of the anode (or electrochemical potential of the anode) with Li intercalated (${µ}_{a}$) is higher than the electrochemical potential of the cathode material (${µ}_{c}$), so initially, it is the cathode that contains lithium [61]. The difference between the two Fermi levels corresponds to the open-circuit voltage (OCV) of the battery by Equation (9), with e being the electron charge [62]:

(9) $$OCV=\frac{{µ}_{a}-{µ}_{c}}{e}$$

During discharge, Fermi levels tend to equalize, so a current flow from the anode to the cathode is accompanied by the deintercalation of Li^+^ from the anode. Moreover, the intercalation into the cathode and the corresponding diffusion through the electrolyte also tend to equalize. On the contrary, during charging, an external potential raises the cathode potential above the anode, promoting the reverse process, the Li^+^ intercalation in the anode and the deintercalation in the cathode [63]. A scheme of the process is given in Figure 3b. In ideal conditions, the open-circuit voltage would correspond to the potential required to charge the battery ($V_{ch}$) and would be the potential exhibited during discharge ($V_{dis}$), following Equations (10) and (11). However, there is a polarization overpotential (η) that increases the former and decreases the latter [62].

(10) $$V_{ch}=OCV+\eta$$

(11) $$V_{dis}=OCV-\eta$$

The overpotential consists of three components [60]: the activation overpotential, the ohmic polarization overpotential, and the concentration polarization overpotential [64]. The first one, the activation overpotential, is related to the activation energy of charge-transfer reactions. The second one, the ohmic polarization overpotential, is due to the internal resistance of the electrodes. Finally, the concentration polarization overpotential is due to limitations in the transport of Li^+^ ions to the active sites of the electrode. On the other hand, considering the specific case of batteries, the energy-storage mechanism renders the magnitude defined as capacitance in the case of supercapacitors obsolete, due to charge storage occurring at a specific potential rather than varying linearly with it [35]. Thus, the specific capacity of a battery (Equation (12)) is defined as the magnitude that measures the charge provided by it (Q), i.e., the integral of the current provided between the start and end of discharge per unit mass or volume (Π) [62,65]. Moreover, it is related to energy density that can be defined as Equation (13) since the charge (Q) is released at constant potential (V).

(12) $$C_{s}=\frac{\int_{0}^{Q}dq}{\Pi }=\frac{\int_{t_{0}}^{t}Idt}{\Pi }$$

(13) $$E_{s}=C_{s}V$$

This release of charge at constant potential gives rise to very characteristic curves, both in cyclic voltammetry tests and galvanostatic charge–discharge tests. In the former, intense peaks appear at the potential of the reaction [45]. On the contrary, these peaks appear as plateaus in galvanostatic charge–discharge tests [45], since as was aforementioned, the charge is released or stored constantly at reaction potentials. In this way, typical CV and GCD curves are shown in Figure 3c. The charge and discharge curves consist of three regions [60], as can be seen in Figure 3d [64]. Taking the example of the discharge curve, first, there is an exponentially decreasing potential region due to the activation overpotential. Next, the plateau appears due to the electrochemical reaction, where the overpotential is mainly due to ohmic polarization. Finally, when there are no active sites left in the electrode, the concentration overpotential causes an exponential drop in potential (*I*·*R* drop). It has to be pointed out that these overpotentials vary with the current density of the process. At a higher current density, higher ohmic polarization overpotential occurs since, according to Ohm’s law, the potential (overpotential in this case) is proportional to the current intensity. The concentration overpotential is also increased, as the kinetics of ion diffusion through the electrolyte limits the intercalation process. Thus, both the potential of the cell and its capacity decrease as the current density of the charge–discharge process increases. In addition, this drop is more dramatic as the internal resistance of the electrodes increases, as can be deduced from Ohm’s law. This performance drop is an important point for industry, so wide ranges of current density are desirable for technological applications. For this reason, low electrical resistivity is an essential condition for Li-ion battery electrode materials.

In conclusion, TMOs are promising materials for Li-ion battery anodes due to their high specific capacity values compared to carbon-based anodes, the most commonly used in conventional batteries [67]. These high-capacity values arise from the lithiation/delithiation mechanism of these materials, which is conversion-based. This mechanism involves reversible reactions with lithium within the crystalline structure, leading to the formation of new species, unlike the intercalation/deintercalation process typical of carbon-based anodes [46,67].

However, the main drawback of TMOs for battery applications is their low level of electrical conductivity, which limits their rate capability, i.e., their performance at high current densities [46,47,48]. Thus, strategies explained in the above section in order to improve the electrical conductivity of TMOs are also widely proposed for the development of TMOs for Li-ion battery anodes, as the combined use with other compounds that exhibit high electrical conductivity, sulfurization or selenization, creation of oxygen vacancies, or the development of multimetallic or multivalent TMOs. Additionally, TMOs for battery applications face a significant challenge: the decrease in capacity over charge–discharge cycles. This decrease is due to the volume expansion of the crystalline structure during lithiation–delithiation processes, leading to material fracture and a loss of electrical contact [61]. In this way, nanostructured and porous TMOs help to mitigate this issue alleviating the stress on the crystalline structure during the volume expansion [67].

## 3. Nanostructured TMOs on Carbon Fiber for Energy-Storage Applications: Obtention Methods

After introducing the requirements for TMOs for supercapacitor electrodes and Li-ion battery anode applications, along with the main reported strategies to overcome their drawbacks, this section will discuss the primary methods for developing TMO coatings on carbon fiber for these applications. In addition to reviewing the state of the art, a comprehensive discussion will be provided.

### 3.1. Solvothermal Synthesis

**Figure 4:**
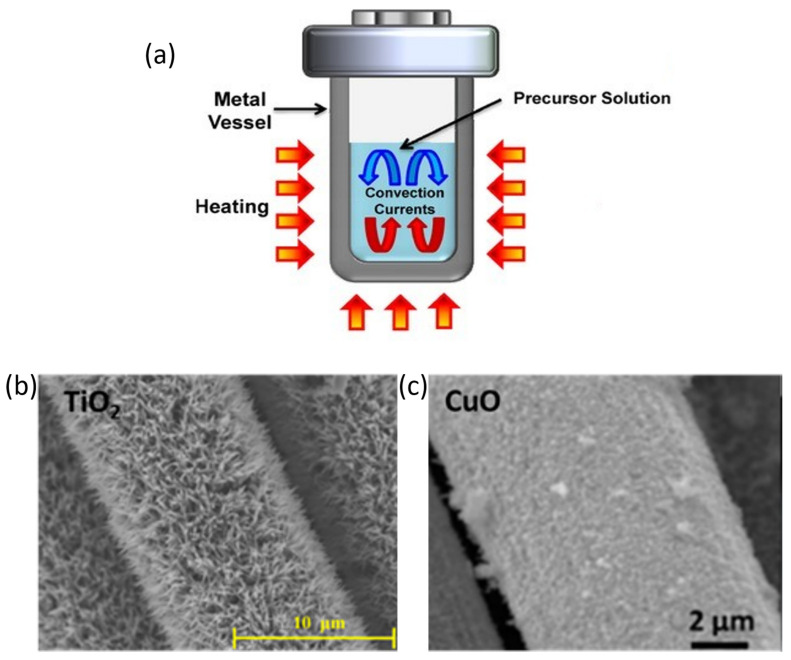
(**a**) Teflon-lined stainless-steel autoclave [65]. Copyright John Wiley and Sons, 2013. (**b**) Different TMOs obtained over carbon fiber: TiO_2_ [99] (Copyright Elsevier, 2016) and (**c**) CuO [96] (Copyright Elsevier, 2016).

Solvothermal, or hydrothermal if the solvent is water, synthesis is the most common route to achieve TMOs [68]. This technique consists of the reaction of a precursor solution under temperature and pressure conditions above the solvent boiling point, generally heating the solution inside a reactor Teflon-lined stainless-steel autoclave, as shown in Figure 4a [69]. The main advantage of this method is the control of the size and shape of the products by varying self-assembly conditions: temperature, time, solvent, or auxiliary reagents. High temperature and pressure favor nucleation, and these factors have a great influence on the size of the final product. On the other hand, the reaction time plays an important role in the size and shape. For example, Ostwald ripening is a commonly proposed model for TMO growth [70]. In that model, smaller spheres redissolve and redeposit on bigger ones, increasing the medium size over time. Moreover, time can promote morphological evolution, for example, from nanosheet to nanoflowers [71], or from spheres to elongated twin-spheres [72].

The shape of the resulting product usually follows the Bravais–Friedel–Donnay–Harker (BFDH) model. The main point in that model is that the growth of the crystal follows a given direction by the surface anisotropy. The system tends to minimize the surface energy of the crystal. Then, the most energetic crystal planes exhibit a higher growth rate and smaller interlayer distances, whereas the less energetic crystal planes show greater stability, lower growth rates, and higher interlayer distances. Thus, Equation (14) defines the growth rate (*R_hkl_*) in a given direction as being inversely proportional to the perpendicular atomic layer spacing (*d_hkl_*) [73].

(14) $$R_{hkl\;}\alpha \;\frac{1}{d_{hkl}}$$

Because of the different physicochemical properties of each solvent increasing the possibility of forming hydrogen bonds or a hydrophobic effect, some crystal faces are favored, promoting different morphologies. Additionally, some solvents, like ethylene glycol (EG) or diethylene glycol (DEG), with more than one functional group, can act as a cross-linker, giving rise to different shapes [74,75], making it possible to control the shape by modulating the ratio between two different solvents [76]. Moreover, because the interaction between the crystal and the solvent depends on the physicochemical properties of the solvent and the crystal surface, the pH also plays a relevant role in the promotion of the growth of certain faces instead of others [74]. In some cases, the use of auxiliary reagents, such as urea, NH_4_F, or HMTA, influences the shape and size too, varying the growth rate of some crystal faces [73,74]. For example, urea decomposes according to the following reactions [74]:

$${CH}_{4}N_{2}O+H_{2}O\;\to 2NH_{3}+{CO}_{2}$$

$$CO_{2}+H_{2}O+NH_{3}\;\to \;{NH}_{4}{HCO}_{3}$$

$${NH}_{4}{HCO}_{3}\;\to \;{NH}_{4}^{+}+{HCO}_{3}^{-}$$

$${HCO}_{3}^{-}\;↔\;H^{+}+{CO}_{3}^{2-}$$

On the one hand, ${CO}_{3}^{2-}$ precipitates with metal cations in the form of carbonates, which posteriorly can be calcinated in the air to obtain final TMO [77,78]. On the other hand, ${CO}_{2}$ creates a reductor atmosphere that avoids metal cation oxidation [79]. In addition, the ${NH}_{4}^{+}$ resulting from urea or HMTA decomposition can act as hydroxyls anions source, which essentially results in some TMO formation [80,81,82]. Other auxiliary reagents are surfactants such as CTAB, which avoids agglomeration, allowing the formation of nanostructured crystals [83].

After the hydrothermal synthesis, thermal treatment in a furnace is a common procedure in order to obtain the final product from other precipitates, to change the morphology, or to increase the crystallinity. These treatments can take place in air, in an inert atmosphere, or involve two steps in different atmospheres [84,85,86,87,88].

Carbon fiber woven is a common substrate for solvothermally obtained TMOs. Several TMOs, such as RuO_2_ [89], MnO_2_ [90,91], Fe_2_O_3_ [92,93], NiO [94], CuO [95,96], ZnO [97,98], TiO_2_ [87,99], Co_3_O_4_ [100], or other spinel structures [88,101,102], have been successfully synthesized on carbon fiber woven through a solvothermal or hydrothermal way for supercapacitors or Li-ion batteries. Some of these coatings are shown in Figure 4b,c. In Figure 4b, TiO_2_ arrays synthesized by Wang and coworkers for a Li-ion anode are presented. They used tetrabutyl titanate as a Ti source and submitted the solution to 180 °C for 12 h. The obtained material displayed a remaining capacity of 188 mAh/g after 500 cycles at a current rate of 0.2 C. On the other hand, Figure 4c shows CuO nanoflowers obtained by Xu and coworkers for supercapacitor electrode applications. In this work, the mass loading of CuO was controlled by the time reaction, reaching the optimum after 12 h of synthesis, when the displayed specific capacitance was 839.9 F/g at a scan rate of 0.1 mV/s, while the bare carbon fiber fabric only exhibited 0.3 F/g at the same conditions. However, there are several works that synthesize these TMOs or even others over other carbon fiber substrates, like carbon fiber paper or carbon fiber mats for energy-storage applications [103,104,105,106,107,108,109,110]. However, these substrates do not exhibit enough mechanical resistance to be used in structural applications, making it necessary to use continuous carbon fiber to achieve this purpose. In order to synthesize TMOs on woven carbon fiber, the carbon fiber is usually functionalized to favor the nucleation on it. There are various methods, such as O_2_-plasma treatment [85] or an autopolymerization coating of dopamine followed by subsequent carbonization [92]. However, the most common surface treatment consists of the partial oxidation of the surface with an acid treatment such as HNO_3_ [89,92,111], HCl [86], or H_2_SO_4_/HNO_3_ mixture in 3:1 proportion in volume [90,94]. These treatments oxidize the carbon fiber surface, generating hydroxyl, carbonyl, and carboxyl groups [112], which can lead to the nucleation of metal cations through electrostatic interactions. However, the more oxidative the treatment is, the more carbon fiber can be deteriorated, decreasing its mechanical properties [112]. Following acid treatment, the fibers must be washed and dried. Another complementary strategy involves seeding metal cations onto the carbon fibers. This process includes repeatedly dipping the fiber into a metal cation solution and subsequently drying it. Typically, a metal cation solution is prepared with a salt of the metal cation whose leaving group is easily evaporated during the drying, for example, acetate. This method creates nucleation points on the carbon fiber, which can lead to the subsequent growth of the TMO during the hydrothermal step [81,113,114].

As it was mentioned in previous sections, electrical conductivity is relevant to achieving a functional Li-ion battery or a supercapacitor. Many efforts have been made in order to improve the TMO’s electrical conductivity and, of course, TMOs on carbon fiber are not an exception. A developed strategy is the doping of TMOs with other compounds that display higher electrical conductivity. Among these compounds, the most usually used are carbon nanostructures such as CNTs [115], graphene [113], rGO [116,117], or carbon dots glucose-derived [118]. In this context, Xie and coworkers demonstrated that graphene@Co_3_O_4_ coatings performed significantly better as Li-ion anodes than Co_3_O_4_ alone. Specifically, the graphene@Co_3_O_4_ coatings exhibited a specific capacity of 391 mAh/g after 300 cycles at 100 mA/g, compared to only 216 mAh/g for Co_3_O_4_ under the same conditions [113].

These compounds or their precursors can be incorporated into the hydrothermal synthesis with other reagents or attached posteriorly through dipping or soaking into a solution containing these compounds. Also, it can be part of a hierarchical structure, which will be discussed later. MXenes, relatively new 2D materials that show exceptional electrical conductivity, have also been reported for this purpose [92]. Another group of materials that can improve the TMO’s electrical conductivity are conductive polymers such as polyaniline (PANI) [119], polypyrrole (PPy) [120,121], or poly(3,4-ethylenedioxythiophene):polystyrenesulfonate (PEDOT:PSS) [102]. For instance, Song and coworkers enhanced the specific capacitance of a positive supercapacitor electrode by 24% at a current density of 1 A/g by spraying a polymer onto carbon fiber coated with hydrothermally obtained CoFe_2_O_4_ [102]. Using these materials, electrons can move along these polymers due to their conjugated systems of π bonds, achieving electrical conductivities on the order of 10^5^ S/cm [122]. The main technique used to coat the CF/TMO with this kind of polymer is electrodeposition. In this process, an electrical current is applied between the substrate and another electrode into a monomer solution to allow polymerization over the substrate, obtaining thin layers over the CF/TMO. However, simpler techniques, such as spraying or immersing, have also been reported [96,114]. Another reported strategy consists of doping with boron through the addition of H_3_BO_3_ in the synthesis step [123]. Besides, other heavier chalcogenides, such as sulfides or selenides, have been synthesized over carbon cloth for these applications, such as MoS_2_ [124], CoS_2_ [125], Ni_3_S_2_ [126], Ni_3_S_4_ [127], NiCo_2_S_4_ [128], CoNi_2_S_4_ [129], and CoFe_2_Se_4_ [130].

In order to achieve larger capacities or better conductivities, hierarchical structures on carbon fiber that involve TMOs are also being studied. These structures pursue taking advantage of synergetic effects between more than one component. Some works generate TMO nanoforms and, over these nanoforms, synthesize another thinner coating of another component, which presents higher energy-storage capability. In this way, the first coating is used to increase the specific area, and the second one is used to improve the capacity or capacitance. To achieve these structures, double solvothermal synthesis has been successfully reported [131,132]. However, solvothermal synthesis can be combined with other synthesis methods, like electrochemical deposition (ECD) [97,133]. On the other hand, in some hierarchical structures, some nanostructures apport not only surface area or energy-storage capability but also electrical conductivity. Similarly, Li and coworkers grew CNTs on carbon fiber via chemical vapor deposition, followed by the synthesis of MnO_2_ or Fe_2_O_3_ on the surface of the CNTs [115]. These materials demonstrated specific capacitances of 367.44 F/g at 2 mA/g and 361.8 F/g at 3 mA/g, respectively, as supercapacitor electrodes.

Finally, some works related to TMO coatings on carbon fiber obtained using the solvothermal method for a supercapacitor electrode and Li-ion battery anode applications are summarized in Table 1 and Table 2, respectively.

### 3.2. MOF-Derived TMOs

In recent years, metal–organic frameworks (MOFs) have been widely studied for different purposes [149,150,151]. These materials consist of crystals compounded by metallic centers linked among them by organic ligands, which exhibit high porosity. Due to their large porosity, which gives rise to a large surface area, MOFs are an interesting material for developing electrodes for energy-storage applications. This surface area results in an increment in the number of electroactive sites and the reactivity [152,153]. Moreover, porosity improves Li^+^ accommodation, improving cyclic performance and ionic diffusion in the case of batteries [18]. However, the electrical conductivity and chemical stability of pristine MOFs are low. Thus, MOFs could be carbonized to achieve higher conductivity values and chemical stability [153,154,155,156,157,158]. During carbonization, metal centers are reduced to M^0^ nanoclusters into a carbon and nitrogen skeleton. Thus, electrical conductivity and chemical stability improve, but some possible electrochemical reactions between metal centers and the electrolyte can disappear, which can decrease the capacitance or capacity. On the other hand, porosity and surface area are reduced due to the structure collapse during carbonization. Another option is to use MOFs as a template to obtain TMOs through oxidation [54,67,152,159,160]; in this case, a nanostructured TMO is obtained. Because of their high porosity, MOF-derived TMOs displayed higher performance as both a supercapacitor electrode and Li-ion battery anode in comparison with bare TMOs. In the case of supercapacitors, the large surface area provides higher specific capacitance values, while, in the case of Li-ion batteries, this large surface reduces Li^+^-diffusion pathways, resulting in a specific capacity increment. Not only that, but cycling stability also increases due to the easier Li^+^ accommodation inside the porous structure [160,161].

**Figure 5:**
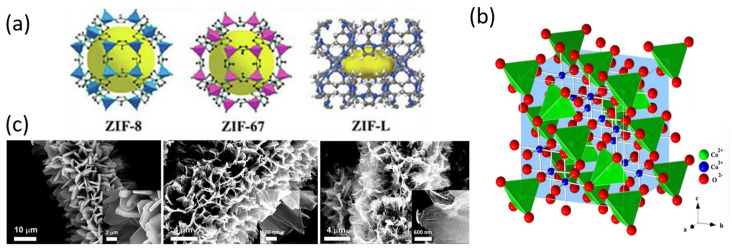
(**a**) ZIF-8, ZIF-67, and ZIF-L structure [168]. Copyright Elsevier, 2021. (**b**) Co_3_O_4_ crystalline structure. (**c**) ZIF-L coating on carbon fiber, MOF-derived NiCo_2_O_4_, and Ni-doped cobalt–cobalt nitride on carbon fiber. Reproduced with permission from [174]. Copyright 2018, American Chemical Society.

Although other MOF coatings have been reported as TMO precursors on carbon fiber [162,163], the most commonly used are zeolitic imidazole frameworks ZIF-8, ZIF-67, and ZIF-L. These MOFs are composed of Zn^2+^, in the case of ZIF-8 and ZIF-L, or Co^2+^, in the case of ZIF-67 and ZIF-L, as a metallic center and 2-methylimidazole as a ligand. The structures of different ZIFs are shown in Figure 5a. As it occurs in solvothermal synthesis, the first step in order to synthesize ZIFs on carbon fiber is to generate hydroxyl, carboxyl, and carbonyl groups on the fiber surface, which act as nucleation points [164,165,166]. Alternatively, another reported strategy to achieve a correct adhesion between ZIFs and carbon fiber is immersing the carbon fiber into a dopamine hydrochloride solution, followed by polymerization into Tris-Cl buffer and subsequent carbonization in order to generate an N-doped super-hydrophilic layer on the carbon fiber surface, which can lead to MOF growth [167].

Both ZIF-8/ZIF-67 or ZIF-L are usually obtained on carbon fiber via coprecipitation of the reactants. However, the obtention of one or the other depends on two main parameters: the solvent and the metal/ligand ratio [168]. In the case of ZIF-L, the plane (0 1 0) energy surface is larger than the (1 0 0) and (0 0 1) plane energy surface, giving rise to layers linked by hydrogen bonds between them, resulting in laminar morphologies, while the ZIF-67 and ZIF-8 structure builds 3D morphologies. Water as a solvent favors ZIF-L obtention due to the fact that, in this case, the hydrogen of the amine group of 2-mIM tends to form hydrogen bonds that link layers. In other solvents like methanol, this hydrogen tends to dissociate; then, the nitrogen attaches to other M^2+^, making 3D morphologies. On the other hand, lower metal/ligand ratios favor ZIF-8 or ZIF-67 obtention due to large concentrations of 2-mIM shifting the dynamic equilibrium and bringing on dehydrogenation. On the contrary, higher metal/ligand ratios favor ZIF-L obtention. Moreover, it is important to control concentration and synthesis time in order to achieve a homogeneous coating.

Next, after MOF synthesis, the samples are submitted to a heat treatment in an oxidative atmosphere in order to obtain the TMO material. In this step, the temperature is the most relevant parameter to take into account, especially in the case of batteries, since it determines the pore volume of the nanosheets. If the temperature is too high, the structure collapses, and volume expansion owing to the lithiation–delithiation process induces mechanical stress into nanosheets, reducing the capacity as the number of cycles increases. This phenomenon was demonstrated by Fu and coworkers for MOF-derived Co_3_O_4_ coatings on carbon fiber [169]. In their work, samples calcinated at 450 °C, 500 °C, and 550 °C exhibited around 5 mAh/g in the initial cycles at 0.5 mA/cm^2^ as Li-ion anodes. However, after 100 cycles, the sample calcinated at 500 °C retained 3.10 mAh/g, while samples calcinated at 450 °C and 500 °C only exhibited 2.30 and 2.05 mAh/g, respectively. However, the optimum temperature reported to obtain the TMO could be detrimental to the mechanical properties of carbon fiber.

The obtained TMOS are Co_3_O_4_ from ZIF-67 and Co-ZIF-L and ZnO from Zn-ZIF-L and ZIF-8. Co_3_O_4_ is one of the most attractive TMOs for energy-storage applications due to its high specific capacity [54]. The crystalline structure of this material is available in Figure 5b. However, other isostructural TMOs in which cobalt occupying tetrahedral sites are substituted by other metals such as Zn, Cu, Ni, or Mn are interesting in order to reduce toxicity and environmental impact [170,171,172,173]. One example of these materials is shown in Figure 5c, where Liu and coworkers synthesized MOF-derived Ni-doped cobalt–cobalt nitride on carbon fiber surface for supercapacitor electrode applications, demonstrating a specific capacitance of 361.93 C/g at a current density of 2 mA/cm^2^ [174]. Another example is the Co_3_O_4_ and ZnCo_2_O_4_ MOF-derived coatings developed by Lim and coworkers. The bimetallic sample exhibited a specific capacity of 40.74 mAh/g at 2 mA/cm^2^, while the monometallic one displayed 12.73 mAh/g under the same test conditions [175]. Not only that, but mixed cobaltite spinels also have displayed better capacities due to the presence of different metals, which favors more chemical reactions, better electrical conductivity, and charge mobility as a result of polaron hopping processes [170,171,172]. The difference in electronegativity and volume between the two metals favors polaron-assisted Li^+^ or OH^−^ diffusion [175]. Mixed cobaltite spinels can be obtained from ZIFs in two main ways. One is by coprecipitation of Co^2+^ and the other metal cation in the corresponding stoichiometric proportion [164], and the other is by the substitution of cations immersing Co-MOF in a solution that contains the other cation [174,176,177].

As has been aforementioned, the main disadvantage of TMOs is their poor electrical conductivity, so different strategies have been reported to increase MOF-derived TMO’s conductivity. One possibility is doping with phosphorus atoms, which act as electron donors, through phosphorylation in an Ar atmosphere with NaH_2_PO_2_ as a P source. In this way, P-Co_3_O_4_ synthesized on carbon fiber by Liu and coworkers displayed a specific capacitance as a supercapacitor electrode, which is 1.52 times greater than that of a Co_3_O_4_ reference sample [166]. Another strategy consists of a partial reduction of TMO with NaBH_4_ to create oxygen vacancies, which not only improve electrical conductivity but also enhance electron and ion transfer between the electrode and the electrolyte [167]. In this way, Dai and coworkers improved the specific capacity of MOF-derived Co_3_O_4_ as a supercapacitor electrode by 2.8 times [167]. Nevertheless, the most common method to obtain these oxygen vacancies is to submit the MOF to an annealing step into an inert atmosphere previously to the necessary calcination to obtain the TMO material. Moreover, ZIFs can be functionalized as chalcogenides in order to reach higher electrical conductivity [167,175,178]. Se and S are softer Pearson’s bases than O, giving rise to less polarized links that favor electron movement. Despite this conductivity improvement, the cycling stability of these compounds is poorer than the corresponding oxides due to a larger expansion volume during lithiation. The obtention of metal sulfurs is usually carried out with thioacetamide (TAA) [179,180,181,182,183] or sulfur powder [184,185] as a sulfidation agent and subsequent thermal treatment, while to produce selenides, selenium powder is the most common selenium source [186,187]. Some works have developed MOF-derived chalcogenides on woven carbon fiber, i.e., Zhang and coworkers synthesized ZIF-derived CoO, CoS, CoSe, and CoTe on carbon fiber cloth, resulting in CoS, which exhibited larger capacitance (3576.0 mF/cm^2^ at a current density of 5 mA/cm^2^) as a supercapacitor electrode [188]. Moreover, Yang and coworkers developed a NiCo-alloy sulfide on a carbon fiber surface from Co(2-mIM) MOF for LIBs, obtaining 213 mAh/g at a current density of 1 A/g [189]. To conclude this section, Table 3 and Table 4 summarize MOF-derived TMOs obtained on carbon fiber for a supercapacitor electrode and Li-ion battery anode applications, respectively.

### 3.3. Electrochemical Deposition (ECD)

**Figure 6:**
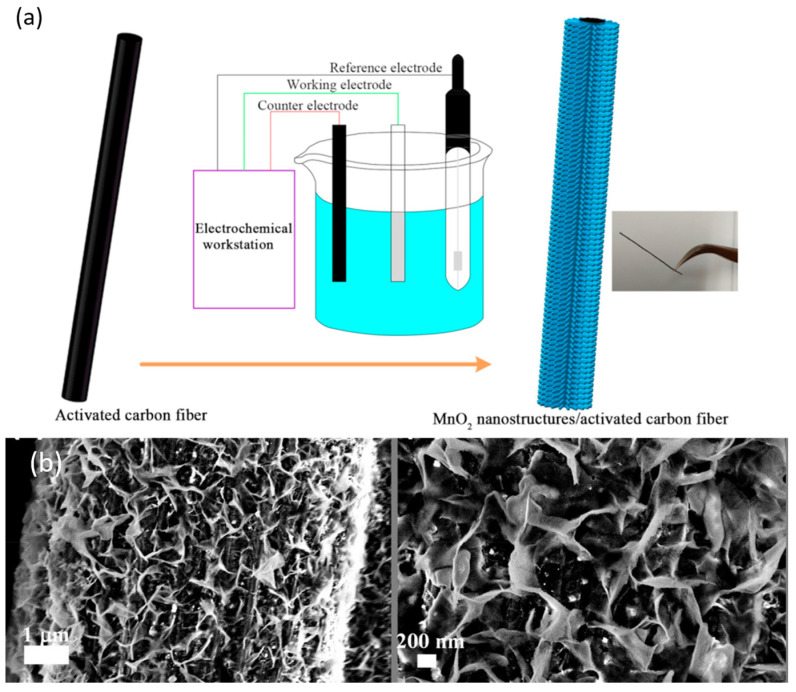
(**a**) Three-electrode system for MnO_2_ electrochemical deposition over carbon fiber [202]. Copyright Elsevier, 2017. (**b**) MnO_2_ coating over carbon fiber. Reproduced with permission from [199]. Copyright 2019, American Chemical Society.

Electrochemical deposition is another reported synthesis route for the obtention of TMOs on carbon fiber. In this technique, carbon fiber is placed as a working electrode in a two- or three-electrode system in which the electrolyte contains the corresponding metal cations, as shown in Figure 6a. A constant potential or a constant current applied promotes the metal cation’s migration to the working electrode and their subsequent oxidation, obtaining the coating. The TMO coating thickness and shape are influenced by the electrodeposition parameters, such as the constant potential or current, the electrodeposition time, and the electrolyte concentration. In addition, several cyclovoltammetry cycles can be applied instead of a constant potential or current to promote migration and oxidation [197].

MnO_2_ is the most reported TMO obtained by electrochemical deposition on carbon fiber due to its high theoretical capacitance, environmental friendliness, natural abundance, and low cost [198]. As previously discussed, increasing the electrical conductivity is essential to improve TMOs’ electrochemical performance for both supercapacitor and battery electrode applications. One strategy can be the modification of the carbon fiber surface. One example is the growth of MnO_2_ nanosheets over carbon fiber coated with AgCNT film [199], obtaining the film via simple immersion in AgCNT ink. Using this method, Ko and coworkers achieved a specific capacitance of 325 F/g at a 1 A/g current density [199]. SEM images of this material are shown in Figure 6b. Other studies have explored hierarchical structures compounded by TMOs and carbon nanocompounds. For instance, Sha and coworkers grew vertical graphenes (VG) on carbon fiber using PECVD to achieve synergetic effects between VG and MnO_2_, achieving an areal capacitance of 30.7 mF/cm^2^ [200]. Another reported strategy involves the polymerization of conductive polymers alongside TMOs, such as the simultaneous electropolymerization of PEDOT:PSS and the electrodeposition of MnO_2_. This approach, used by Wang and coworkers, resulted in an electrode with an areal capacitance of 386 mF/cm^2^ at a current density of 1 mA/cm^2^ [201].

In addition to MnO_2_, other TMOs obtained by electrodeposition on carbon fiber substrates are Fe_2_O_3_ [201], V_2_O_5_ [203], Co_3_O_4_ [204], NiCo_2_O_4_ [205], and NiO [206]. However, only a few of these works involve a strategy to improve the electrical conductivity. Li and coworkers modified carbon fiber with Ni via electrodeposition previous to NiCo_2_O_4_ electrodeposition [207]. Additionally, a hierarchical structure in which NiO coats CNT grown on carbon fiber paper via CVD was proposed. In this work, the proposed electrode material by Zhang and coworkers demonstrated a specific capacitance of 1317 F/g at 1.2 A/g [206]. On the other hand, conductor polymers like PPy have been reported to improve conductivity in this kind of coating [205]. Moreover, other transition metal chalcogenides, which present better electrical conductivity, have been synthesized on carbon fiber cloth via electrochemical deposition as CoNi_2_S_4_ [129]. Not only that, as mentioned in Section 3.1, electrodeposition can be combined with other synthesis routes to obtain hierarchical structures that involve more than one TMO, or a TMO and a derivative like Co_9_S_8_@NiCo_2_O_4_ [197], NiCo_2_O_4_@Ni(OH)_2_ [208], MoO_2_@MnO_2_ [209], or CuO@MnO_2_ [210]. As a summary, reported TMOs obtained on carbon fiber by ECD for supercapacitor electrode purposes are presented in Table 5.

Although TMOs obtained by electrodeposition are more often used for supercapacitors than batteries, TMO obtained in this way can also be employed as Li-ion battery anodes, i.e., Qiu and coworkers developed Fe_3_O_4_ nanotube arrays over a carbon fiber cloth via a ZnO sacrificial template obtained by electrodeposition. As Li-ion battery anodes, it exhibited a specific capacity of 930 mAh/g at a current density of 2 mA/g and a capacity retention of 73% after 200 GCD cycles [212].

## 4. Conclusions and Outlook

Currently, the development of all-solid-state energy-storage devices, such as Li-ion batteries and supercapacitors, has become a key objective for both researchers and industry. The interest in flexible devices stems not only from the latest trend in the electronic devices market but also from advancements in wearable biomedical devices. These new devices require energy-storage systems that are preferably flexible to ensure wearability.

On the other hand, the interest in structural devices interest is related to the development of multifunctional materials that can reduce the mass of vehicles. Electric vehicles, in particular, could greatly benefit from these multifunctional materials since their batteries are especially heavy. Partial or total substitution of traditional batteries with structural energy storage materials could mean an important improvement in autonomy, which currently is their main drawback to significantly enhancing vehicle range, addressing one of the main drawbacks of current electric vehicles.

Thereby, flexible and structural energy-storage materials are crucial for the sustainability of urban environments and transportation, as well as for the advancement of health monitoring technologies. However, the achievement of these devices is still far from having been accomplished, especially in the case of the structural ones. In this context, the use of composites has emerged as some of the most promising materials for these purposes due to the high potential of carbon fiber as a reinforcement and substrate that provides mechanical strength and electrical conductivity. Nevertheless, the carbon fiber electrochemical performance should be improved in order to become a functional energy-storage device component. This is where TMO coatings emerge as a possible solution due to their high theoretical capacitance as a supercapacitor electrode and capacity as a Li-ion battery anode. In this way, some techniques have been reported to obtain these coatings, with the most common being the following three:

- Solvothermal/hydrothermal synthesis: This technique offers the significant advantage of achieving a wide range of transition metal oxide compositions and nanostructure morphologies by varying only a few parameters. As Li-ion anodes, different transition metal oxides yield different theoretical capacities due to various conversion reactions. Additionally, different morphologies can affect cyclability. On the other hand, as supercapacitor electrodes, diverse transition metal oxides provide varying pseudocapacitive reactions and electrical conductivities, with different nanostructured morphologies offering different surface areas, a crucial factor for supercapacitor electrode capacitance. Consequently, the extensive possibilities offered by hydrothermal synthesis make it the most developed technique for these applications while also opening up a wide range of potential optimizations and new materials for future research.
- Metal–organic framework-derived synthesis: Using MOFs as precursors to obtain transition metal oxides results in more porous coatings. Increased porosity enhances the surface area of transition metal oxides, leading to higher supercapacitor electrode capacitance. On the other, for Li-ion battery anodes, increased porosity improves capacity and cyclability. However, a major drawback of this technique is that MOF-derived coatings are primarily limited to the zeolitic–imidazole frameworks series, resulting in a limited variety of available transition metal oxide structures. Therefore, the primary goal of this technique should be to develop new MOF coatings on the carbon fiber to expand the range of available materials.
- Electrochemical deposition: This is the least developed of the three techniques, particularly for Li-ion battery applications. Consequently, it presents significant potential for future research. One advantage of electrochemical deposition is the ability to deposit transition metal oxide alongside other compounds, such as conductive polymers, which can act synergistically with the oxides.

On the other hand, despite the synthesis method, the main problem of TMOs is their low electrical conductivity. Therefore, it is essential not only to control the composition and morphology of these coatings but also to develop strategies to improve their electrical conductivity. Some reported strategies are the combination of TMOs with conductive materials such as carbon-based nanostructures or conductive polymers, the creation of oxygen vacancies, the dopping with donor elements as P, the sulfurization or selenization, or the synthesis of the multivalence or multimetallic transition metal oxides. Hence, the development of these strategies on TMO coatings on carbon fiber is as crucial as the development of new coating compositions and morphologies. Moreover, the similar requirements for both supercapacitor electrodes and Li-ion battery anodes suggest that advances in materials for supercapacitor electrodes, which have been more extensively developed and studied, could be effectively applied to Li-ion battery applications.

Finally, some recent studies have used hierarchical structures as carbon fiber coatings for supercapacitor electrodes and Li-ion battery anode applications. This paves the way for a new generation of coatings that can take advantage of the synergy between more than one transition metal oxide or even between transition metal oxides and other materials, such as CNTs grown by CVD or vertical graphene.

## Figures and Tables

**Table 1:** TMOs on carbon fiber for supercapacitor electrode applications.

Material	Other Reactants/ Solvents	Solvothermal Treatment	Post-Treatment	Capacitance	Current Density	Capacitance Retention	Cycles	Electrolyte	Reference
MnO_2_ nanosheets + CNTs	-/H_2_O	180 °C 2 h	-	367.44 F/g	2 mA/cm^2^	97.43%	10,000	Na_2_SO_4_ 1 M	[115]
Fe_2_O_3_ nanodots + CNTs	Urea/H_2_O	130 °C 8 h	-	361.8 F/g	3 mA/cm^2^	98.07%	10,000	Na_2_SO_4_ 1 M	[115]
MnO_2_ nanowhiskers	-/H_2_O	160 °C 3 h	-	490 F/g	1 A/g	87.5%	10,000	KOH 6 M	[107]
B-doped MnO_2_ worm-like films	H_3_BO_3_/H_2_O	80 °C 8 h	-	210 F/g	5 A/g	80.4%	900	Na_2_SO_4_ 0.5 M	[123]
MnO_2_ nanowires	-/H_2_O	140 °C 3 h	-	3.88 F/cm^2^	5 mA/cm^2^	91.5%	3000	Na_2_SO_4_ 1 M	[91]
MnO_2_ + PCC	-/H_2_O	90 °C 6 h	-	1065 mF/cm^2^	1 mA/cm^2^	-	-	LiCl 5 M	[92]
Fe_2_O_3_ nanorod arrays + MXenes	Na_2_SO_4_/H_2_O	120 °C 8 h	450 °C 2 h in N_2_ + dipping in Mxene solution	725 mF/cm^2^	1 mA/cm^2^	88.6%	10,000	LiCl 5 M	[92]
MnO_2_ nanocrystals	NaNO_3_/H_2_O	100 °C 24 h	-	1.66 F/cm^2^	2 mA/cm^2^	88.6%	5000	Na_2_SO_4_ 1 M	[93]
NiO nanosheets	-/H_2_O	80 °C 6 h	Soaking in KOH 2 M	842 mF/cm^2^	1 mA/cm^2^	-	-	KOH 3 M	[134]
NiO + PANI	Urea/H_2_O	120 °C 6 h	300 °C 3 h + PANI ECD	192.3 F/g	0.5 A/g	72%	4500	Na_2_SO_4_ 0.5 M	[119]
Co_3_O_4_ nanorod bundle arrays + PPy	Urea + NH_4_F/H_2_O	120 °C 8 h	Annealing 350 °C 3 h + immersing in PPy solution	6.67 mF/cm^2^	2 mA/cm^2^	~100%	2000	KOH 1 M	[120]
Co_3_O_4_ flakes + MnO_2_ sheets	I (Urea) /H_2_O	I (180 °C 2 h) + II (160 °C 2 h)	-	1396 F/g	0.3 A/g	98%	5000	KOH 1 M	[132]
Co_3_O_4_ nanowires/nanoflowers	Urea/EtOH	180 °C 12 h	300 °C 3 h	7.8 mF/cm^2^	0.2 mA/cm^2^	54%	5000	KOH 3 M	[100]
Co_3_O_4_ + RGO	-/EtOH	140 °C 12 h	Soaking in GO solution + 500 °C 2 h in Ar	5.91 F/cm^2^	4 mA/cm^2^	58.64%	5000	KOH 1 M	[135]
NiCo_2_O_4_ nanowire arrays + PPy	Urea/H_2_O	80 °C 6 h	300 °C 2 h in air + PPy ECD	1.44 F/cm^2^	2 mA/cm^2^	85%	5000	KOH 3 M	[121]
NiCo_2_O_4_ nanowires + rGO	GO + urea /H_2_O + EtOH	120 °C 8 h	350 °C 2 h	931.7 F/g	1 A/g	-	-	KOH 3 M	[116]
NiCo_2_O_4_ nanospikes + rGO	Urea + NH_4_F/H_2_O	150 °C 5 h	Annealing 300 °C 3 h	1338 mF/cm^2^	3 mA/cm^2^	88.2%	10,000	KOH 3 M	[117]
NiCo_2_O_4_ rods + Ni-Co LDH	Urea + NH_4_F/H_2_O	120 °C 6 h	Ni-Co LDH ECD	4901.8 mF/cm^2^	2 mA/cm^2^	86.7%	5000	KOH 1 M	[133]
CuO nanosheets	NaOH /H_2_O + EG	120 °C 12 h	-	1.64 F/g	40 mA/g	99.45%	10,000	Na_2_SO_4_ 1 M	[95]
CuO nanoflowers	NaOH + urea/EtOH	373 K 12 h	-	839.9 F/g	1 mV/s	91%	2000	KOH 6 M	[96]
ZnO + Ni-Co-S core–shell nanorods	HMTA + ammonia/H_2_O	90 °C 24 h	Ni-Co-S ECD	1302.5 F/g	1 A/g	65%	5000	KOH 1 M	[97]
ZnO nanoflowers	NaOH + CTAB/H_2_O	100 °C 10 h	350 °C 2 h	201.25 F/g	1 A/g	90.31%	3000	KOH 1 M	[136]
ZnO nanotubes	HMTA/H_2_O	100 °C 8 h	Dipping in KOH solution	14.25 F/g	1 mV/s	93.2%	2500	IL (15%)/LiS (5%)/PES (78%)/PANI (2%)	[98]
TiO_2_	-/C_3_H_8_O_3_ + C_2_H_h_O	150 °C 15 h	320 °C 4 h in air + reduction in NaBH_4_ solution	115.3 F/g	1200 mA/g	98.7%	10,000	Na_2_SO_4_ 1 M + Fe(CN)6^3−/4−^	[87]
ZnCo_2_O_4_ nano polyhedra	-/H_2_O + EG	180 °C 20 h	350 °C 2 h in air	2643.66 F/g*	2 A/g	96.54%	5000	KOH	[88]
CoFe_2_O_4_ microspheres	-/H_2_O + glycol	200 °C 8 h + 200 °C 4 h	300 °C 3 h	393 mF/cm^2^	1 A/cm^2^	95.8%	2000	KOH 3 M	[101]
CoFe_2_O_4_	-/H_2_O + EG	180 °C 24 h	350 °C 3 h + spraying PEDOT:PSS	472.5 F/g	1 A/g	~90%	5000	Na_2_SO_4_ 1 M	[102]
MnO_2_-Mn_3_O_4_	-/H_2_O // -/EtOH + H_2_O + EG	160 °C 6 h + 180 °C 5 h	-	1708.8 F/g	1 A/g	91.9%	6000	Saturated KCl	[137]
CuO-MOF nanosheets	BTC/H_2_O + EtOH	150 °C 15 h	-	953 F/g	400 mA/g	97.5%	10,000	Na_2_SO_4_ 1 M + Fe(CN)6^3−/4−^ 0.05 M	[138]
Fe_2_O_3_ nanorod arrays	Urea/H_2_O	100 °C 12 h	500 °C 3 h in air	976.4 mF/cm^2^	4 mA/cm^2^	104%	10,000	KOH 1 M	[139]
NiO film	C_6_H_15_NO_3_/ H_2_O	200 °C 1 h	300 °C 2 h in air	207 F/g	0.5 A/g	95%	2000	KOH 3 M	[140]
Mn_3_O_4_@ NPC	Chitosan/ H_2_O	180 °C 10 h	600 °C 1 h in N_2_	256.8 F/g	1 A/g	97.3%	5000	Na_2_SO_4_ 1 M	[141]
CuO nanoparticles	-/ H_2_O	80 °C 2 h	-	5.42 F/g	10 mV/s	-	-	KCl 3 M	[142]
V_2_O_5_/SnO_2_	CTAB/EtOH + H_2_O	180 °C 12 h	-	151.3 F/g	1 A/g	92.1%	10,000	KOH 4 M	[143]
FeCo_2_O_4_ nanoparticles	Urea + NH_4_F/EtOH + H_2_O	140 °C 7 h	400 °C 2 h	912 F/g	2 A/g	90%	10,000	KOH 1 M	[144]

**Table 2:** TMOs on carbon fiber for Li-ion battery anode applications.

Material	Other Reactants/ Solvent	Solvothermal Treatment	Post-Treatment	Reversible Capacity	Current Density	Cycles	Reference
NiO nanosheets + CD	HMTA/H_2_O	120 °C 10 h	900 °C 90 min in N_2_ +500 °C 3 h in air	2.91 mAh/cm^2^	3 mA/cm^2^	250	[118]
NiO	NH_4_F + urea/H_2_O + isopropanol	180 °C 8 h	3 h in Ar	171 mAh/g	100 mA/g	60	[94]
MnO_2_	-/H_2_O + EG	3 h stirring + 30 min ultrasonication	350 °C 40 min	648 mAh/g	100 mA/g	150	[90]
RuO_2_ nanoparticles	-	Soaking	500 °C 15 min in air	1 mAh/g	1 mA/cm^2^	3500 h	[89]
TiO_2_ + Fe_2_O_3_ nanotube arrays	I (glycerinum/EtOH) + II (ammonia/H_2_O)	I (175 °C 20 h) + II (95 °C 4 h)	I (350 °C 1 h in N2) + II (450 °C 2 h)	896 mAh/g	200 mA/g	200	[111]
TiO_2_ arrays	Glycerinum/EtOH	180 °C 4 h	-	188 mAh/g	0.2 C	500	[99]
MoS_2_ + Fe _3_ nanosheets	I (l-cysteine + glucose/H_2_O + DMF) + II (HMTA; ammonia/H_2_O)	I (200 °C 12 h) + II (85 °C 24 h)	I (700 °C 2 h in Ar) + II (self-sacrificing template + 550 °C 5 h in air)	1162.3 mAh/g	0.5 A/g	200	[131]
VO_2_ nanobelt arrays	H_2_O_2_ (30%) + oxalic acid/H_2_O + EtOH	180 °C 3 h	-	130 mAh/g	1000 mA/g	200	[145]
V_2_O_5_ nanoflake arrays	H_2_O_2_ (30%) + oxalic acid/H_2_O + EtOH	180 °C 3 h	-	230 mAh/g	2000 mA/g	300	[146]
Co_3_O_4_ nanoparticles + graphene	-/H_2_O + EtOH	180 °C 2 h	700 °C 2 h in Ar + 250 °C 2 h in air	391 mAh/g	100 mA/g	300	[113]
Co_3_O_4_ + ZnO nanosheets	Urea + CTAB/H_2_O + EtOH + EG	120 °C 10 h	450 °C 2 h in Ar + 200 °C 8 h in air	1102.5 mAh/g	0.5 A/g	400	[85]
ZnCo_2_O_4_ urchins	Urea/H_2_O	200 °C 12 h	400 °C 2 h	1180 mAh/g	0.2 C	100	[84]
NiCo_2_O_4_ nanowire arrays	Urea + NH_4_F/H_2_O	100 °C 10 h	400 °C 2 h in N_2_	1085.5 mAh/g	500 mA/g	100	[86]
ZnCo_2_O_4_	Urea + NH_4_F/H_2_O	120 °C 5 h	400 °C 2 h in air	787.2 mAh/g	100 mA/g	150	[147]
Mn_3_O_4_	Oxalic acid/H_2_O	200 °C 10 h	400 °C 3 h in air	610.5 mAh/g	100 mA/g	150	[148]

**Table 3:** MOF-derived TMOs obtained on carbon fiber for supercapacitor electrode applications.

MOF Precursor	TMO	MOF Obtention	Heat Treatment	Capacitance/ Capacity	Current Density	Capacity Retention	Cycles	Electrolyte	Reference
ZIF-67	Co_3_O_4_ nanosheets with O vacancies	Coprecipitation + reduction with NaBH_4_	500 °C 30 min in N_2_ + 2 h 350 °C 2 h in air	414 C/g	1 A/g	73.9%	15,000	LiOH 2 M	[167]
ZIF-67	S-Co_3_O_4_ polyhedrons	Coprecipitation	2.5 h in autoclave with TAA solution	970 mF/cm^2^	5 mA/cm^2^	67.7%	5000	KOH 2 M	[190]
Co(2-mIM) MOF	Co_3_O_4_ nanosheets	Coprecipitation	500 °C 1 h in N_2_ + 350 °C 2 h in air	12.731 mAh/g	2 mA/cm^2^	89.6%	2000	KOH 3 M	[175]
CoZn(2-mIM) MOF nanosheets	ZnCo_2_O_4_ nanosheets	Coprecipitation	500 °C 1 h in N_2_ + 350 °C 2 h in air	40.741 mAh/g	2 mA/cm^2^	95.1%	2000	KOH 3 M	[175]
Co(2-mIM) MOF nanosheets	NiCo_2_O_4_ nanosheets	Coprecipitation + ion exchange (Ni)	350 °C 2 h in air	1055.3 F/g	2.5 mA/cm^2^	-	-	KOH 2 M	[177]
ZIF-L	Ni-doped Co_2_N nanosheets	Coprecipitation + ion exchange (Ni)	350 °C 2 h in air + 350 °C 2 h in NH_3_	361.93 C/g (Ni-doped Co_2_N)	2 mA/cm^2^ (Ni-doped Co_2_N)	82.4% (Ni-doped Co_2_N)	5000 (Ni-doped Co_2_N)	KOH 1 M	[174]
Co(2-mIM) MOF	P-Co_3_O_4_ nanosheets	Coprecipitation	700 °C 2 h in Ar/H_2_ + 350 °C 2 h in air + 350 °C 2 h in air with NaH_2_PO_2_	337 mF/cm^2^	1 A/cm^2^	97.6%	10,000	KOH 2 M	[166]
CoZn(2-mIM) MOF nanosheets	ZnCo_2_O_4_ nanosheets	Coprecipitation + ion exchange (Zn)	350 °C 2 h in air	536.8 mF/cm^2^	2.5 mA/cm^2^	-	-	KOH 2 M	[176]
CoMn(2-mIM) MOF	MnCo_2_O_4_ nanosheets	Coprecipitation + ion exchange (Mn)	350 °C 2 h in air	515 mF/cm^2^	2.5 mA/cm^2^	95.5%	20,000	KOH 2 M	[176]
Co(2-mIM) MOF	Co_3_O_4_ nanosheets	Coprecipitation	500 °C 1 h in N_2_ + 350 °C 2 h in air	225 mF/cm^2^	10 mA/cm^2^	-	-	KOH 2 M	[178]
Co(2-mIM) MOF	P-MnCo_2_O_4_ nanotube arrays	Coprecipitation + ion exchange	350 °C 2 h in air with NaH_2_PO_2_	996.7 F/g	1 A/g	85.7%	3000	KOH 6 M	[191]
ZIF-67	Co_3_O_4_ nanosheets	Coprecipitation	290 °C 1 h in air	251 F/g	1 A/g	90%	5000	KOH 1 M	[192]
ZIF-67	Walnut-like CoO nanosheets	Coprecipitation	250 °C 2 h in air	842 F/g	1 A/g	96.4	10,000	KOH 6 M	[193]

**Table 4:** MOF-derived TMOs obtained on carbon fiber for Li-ion battery anode applications.

MOF Precursor	TMO	MOF Obtention	Heat-Treatment	Reversible Capacity	Current Density	Cycles	Reference
Co(2-mIM) MOF	Co_3_O_4 _ nanosheets	Coprecipitation	500 °C 1 h in air	3.1 mAh/cm^2^	0.5 mA/cm^2^	100	[169]
CoZn(2-mIM) ZIF	ZnCo_2_O_4_ nanosheets	Coprecipitation	450 °C 2 h in air	3.01 mAh/cm^2^	0.24 mA/cm^2^	100	[194]
Co(2-mIM) ZIF	Co_3_O_4_ nanosheets	Coprecipitation	450 °C 2 h in air	1.93 mAh/cm^2^	0.24 mA/cm^2^	100	[194]
CoZn(2-mIM) MOF	ZnCo_2_O_4_ nanosheets	Coprecipitation + ion exchange	350 °C 2 h in air	1376 mAh/g	1 A/g	200	[176]
CoMn(2-mIM) MOF	MnCo_2_O_4_ nanosheets	Coprecipitation + ion exchange	350 °C 2 h in air	1289 mAh/g	1 A/g	200	[176]
Co(2-mIM) MOF	ZnCo_2_O_4_ polyhedrons	Coprecipitation	400 °C 3 h in N_2_	463 mAh/g	50 mA/g	100	[164]
Co(2-mIM) MOF	Co_3_O_4_ nanosheets	Coprecipitation	300 °C 1 h in air	-	-	-	[195]
Zn(2-mIM) MOF	ZnO nanosheets	Coprecipitation	300 °C 1 h in air	-	-	-	[195]
ZIF 67	Co_3_O_4 _ polyhedrons	Coprecipitation	400 °C 1 h in air	420 mAh/g	100 mA/g	300	[165]
ZIF-8	ZnO polyhedrons	Coprecipitation	450 °C 1 h in air	510 mA/g	100 mA/g	300	[196]

**Table 5:** TMOs obtained on carbon fiber by ECD for supercapacitor electrode applications.

Material	ECD Electrolyte	Current Density/ Potential/ CV Potential Window	Time/Cycles (Scan Rate)	Capacitance	Current Density	Capacitance Retention	Cycles	Electrolyte	Reference
VG/MnO_2_ nanoflowers	Na_2_SO_4_ + MnSO_4_	0.4 mA/cm^2^	40 min	30.7 mF/cm^2^	0.5 mA/cm^2^	93.8	2000	Na_2_SO_4_ 1 M	[200]
Silane-treated MnO_2_	Mn(CH_3_CO_2_)_2_	1 mA/cm^2^	10 min	5.68 mF/cm^2^	2.5 µA/cm^2^	92	5000	EP-IL	[211]
AgCNTs/MnO_2_ nanosheets	Na_2_SO_4_ + MnSO_4_	−1.8 V	10 min	325 F/g	1 A/g	82.2	5000	Na_2_SO_4_ 0.5 M	[199]
MnO_2_/PEDOT	Mn(CH_3_CO_2_)_2_*4H_2_O +PEDOT:PSS	7.0 V	-	386 mF/cm^2^	1 mA/cm^2^	98.4	1800	LiCl 1 M	[201]
Fe_2_O_3_	Fe(NO_3_)_2_*9H_2_O + Na_2_SO_4_	7.0 V	-	315 mF/cm^2^	1 mA/cm^2^	94.6	10,000	LiCl 1 M	[201]
V_2_O_5_	VOSO_4_*xH_2_O	1.2 V	30 min	354 mF/g	1 mA/cm^2^	-	-	Na_2_SO_4_ 1 M	[203]
Co_9_S_8_/NiCo_2_O_4_ core–shell nanoneedles	Co(NO_3_)_2_ + Ni(NO_3_)_2_	−0.6 → −1.2 V	5 (20 mV/s)	1022.5 F/g	1 A/g	88.9	6000	KOH 6 M	[197]

## Data Availability

Not applicable.
